# Supplementary material for: Wavevector multiplexed atomic quantum memory via spatially-resolved single-photon detection
Source: Nat Commun. 2017 Dec 15;8:2140. doi: 10.1038/s41467-017-02366-7 (PMC5732182; doi:10.1038/s41467-017-02366-7)
Supplement: Supplementary file 1 — Supplementary Information [file 41467_2017_2366_MOESM1_ESM.pdf]

SUPPLEMENTARY FIGURES

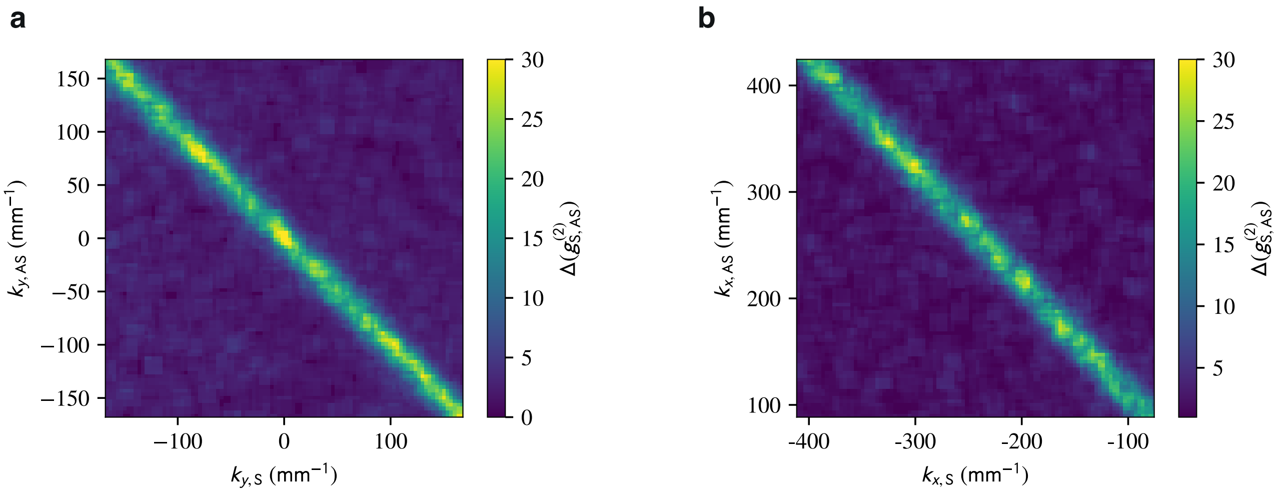

Supplementary Figure 1. **Standard deviation of results for $g^{(2)}_{\mathrm{S,AS}}$.** Panels **(a-b)** correspond to maps from panels a-b in Fig. 4 of the main manuscript. These inferred errors are taken as one standard deviation of a set of results from 25 different sets of regions selected in conjugate columns oriented in y-direction (x-direction) for a (b).

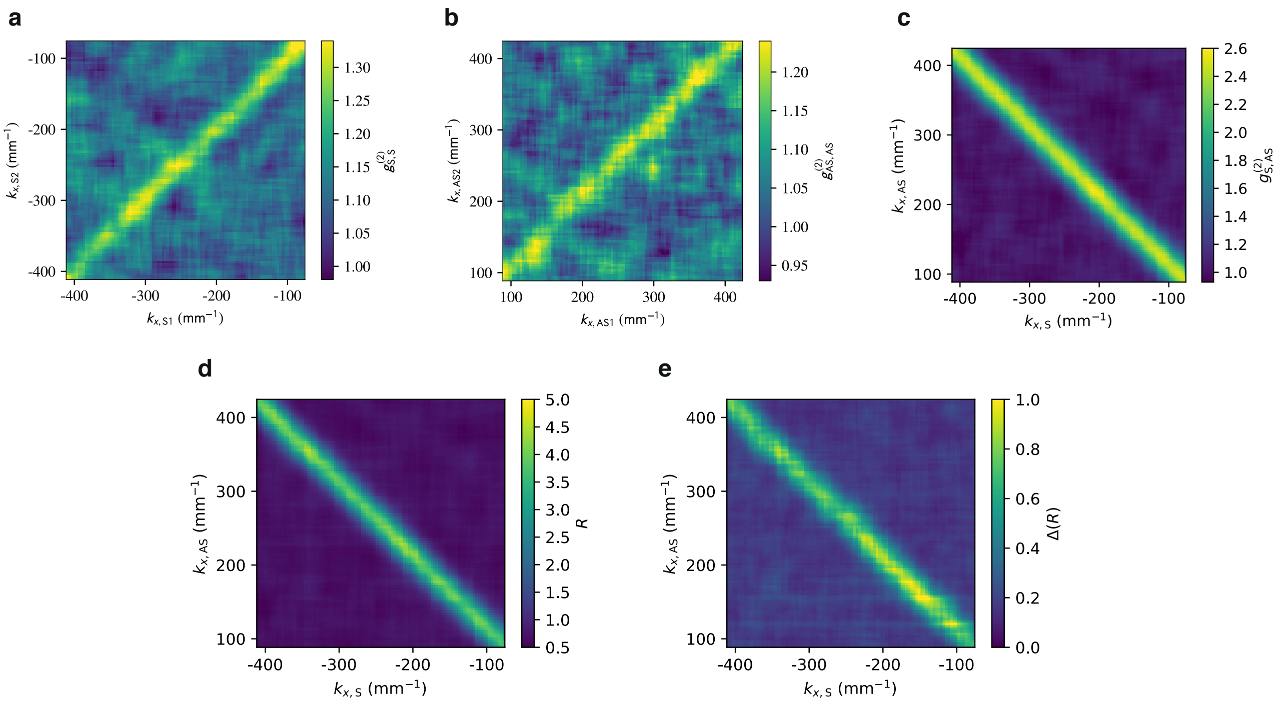

Supplementary Figure 2. **Results of the autocorrelation measurements.** **(a-c)** Results of the measurement of $g^{(2)}_{\mathrm{S,S}}$, $g^{(2)}_{\mathrm{AS,AS}}$ and $g^{(2)}_{\mathrm{S,AS}}$, respectively. The measurement was performed using the method described in Methods section of the main manuscript. To obtain better statistics a higher number of generated photons was used than in other measurements, and thus the second-order cross-correlation function is relatively low. Additionally, a larger analysis region with side length $\kappa = 25.2\ \mathrm{mm}^{-1}$ was used, resulting in visibly wider diagonal correlation. By averaging diagonal, correlated region we found mean values of $g^{(2)}_{\mathrm{S,S}} = 1.29 \pm 0.04$, $g^{(2)}_{\mathrm{AS,AS}} = 1.18 \pm 0.04$ and $g^{(2)}_{\mathrm{S,AS}} = 2.45 \pm 0.04$. For a set of uncorrelated regions we found $g^{(2)}_{\mathrm{S,S}} = 1.07 \pm 0.04$, $g^{(2)}_{\mathrm{AS,AS}} = 1.04 \pm 0.03$ and $g^{(2)}_{\mathrm{S,AS}} = 1.02 \pm 0.05$. These values are consequently higher than the expected value of 1 in the ideal case scenario, which is due to significant classical long-term fluctuations during the measurement. **(d)** Inferred value o R demonstrating significant violation of the Cauchy-Schwartz inequality at the diagonal. Respective standard deviations of the values of R are presented in **(e)**. By averaging the diagonal values we find $R = 4.0 \pm 0.2$. For uncorrelated regions the value is $R = 0.68 \pm 0.06$. Errors correspond to one standard deviation. Analogous results were obtained for the y-dimension.

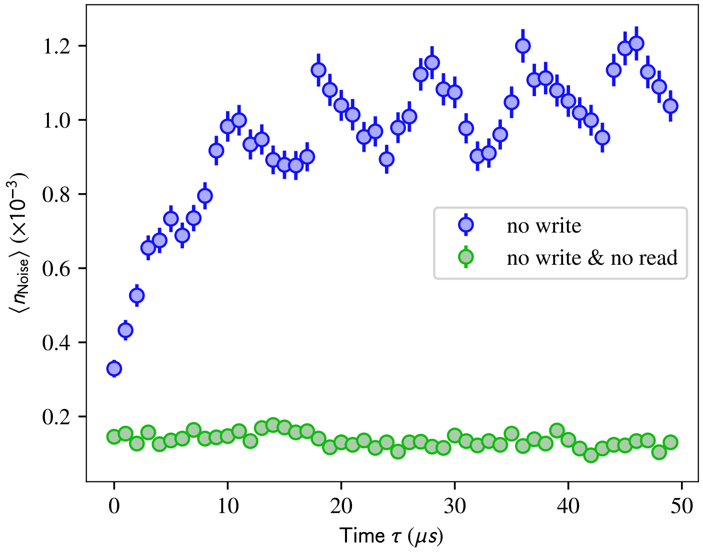

Supplementary Figure 3. **Noise level as a function of storage time.** The mean number of registered noise photons in the AS arm with (blue dots) and without (green dots) the read laser. The write laser was off in both cases. The noise level without the read laser corresponds to mostly dark counts and residual stray light. With the read laser we observe fluorescence of atoms residing in $F = 2$ manifold of the ground state. The initial increase of this fluorescence is due to influx of room-temperature atoms present in the vacuum chamber into the interaction region. The optical pumping ensures that at the zero storage time the fluorescence is minimized. We attribute the oscillation in the noise photons signal to partial spin polarization of room-temperature atoms. Errorbars (one standard deviation) correspond to statistical uncertainty calculated assuming Poissonian distribution of photon counts. Statistical uncertainty for the second case (green circles) is negligible.
